# p53 overexpression is associated with cytoreduction and response to chemotherapy in ovarian cancer

**DOI:** 10.1038/sj.bjc.6690756

**Published:** 1999-10

**Authors:** G Ferrandina, A Fagotti, M G Salerno, P G Natali, M Mottolese, F Maneschi, A De Pasqua, P Benedetti-Panici, S Mancuso, G Scambia

**Affiliations:** Department of Obstetrics and Gynecology, Catholic University of the Sacred Heart, L.go Gemelli 8, Rome, 00168, Italy; Department of Human Pathology, Istituto Regina Elena, Rome, Italy; Libero Istituto Universitario Campus Biomedico, Rome, Italy

**Keywords:** p53, ovarian cancer, cytoreduction, response to chemotherapy

## Abstract

The aim of this study was to assess the association of p53 status with primary cytoreduction, response to chemotherapy and outcome in stage III–IV primary ovarian cancer patients. Immunohistochemical analysis of p53 was performed on formalin-fixed, paraffin-embedded specimens from 168 primary ovarian carcinomas by using the DO-7 monoclonal antibody. p53 nuclear positivity was found in 84 out of 162 (52%) malignant tumours. A higher percentage of p53 nuclear positivity was observed in patients with advanced stage of disease than in stage I–II (57% vs 23% respectively; *P* = 0.0022) and in poorly differentiated versus well/moderately differentiated tumours (59% vs 32% respectively; *P* = 0.0038). The multivariate analysis aimed to investigate the association of FIGO stage, grade and p53 status with primary cytoreduction in 136 stage III–IV patients showed that stage IV disease may influence the possibility to perform primary cytoreduction in ovarian cancer patients. p53-positivity also maintained a trend to be associated with poor chance of cytoreduction. In patients who underwent pathologic assessment of response, cases who did not respond to chemotherapy were much more frequently p53-positive than p53-negative (86% vs 14% respectively; *P* = 0.012). Moreover, patients with stage III disease and < 2-cm residual tumour were more likely to respond to treatment. In multivariate analysis, FIGO stage and p53 expression were independently correlated with pathologic response to chemotherapy. Time to progression and survival rates were shown not to be different in p53-positive versus p53-negative patients. © 1999 Cancer Research Campaign


*British Journal of Cancer* (1999) **81**(4), 733–740
© 1999 Cancer Research Campaign
Article no. bjoc.1999.0756

### p53 overexpression is associated with cytoreduction and response to chemotherapy in ovarian cancer

**G Ferrandina[1], A Fagotti[1], MG Salerno[1], PG Natali[2], M Mottolese[2], F Maneschi[3], A De Pasqua[1], P Benedetti-Panici[3], S Mancuso[1] and G Scambia[1]**

[1]Department of Obstetrics and Gynecology, Catholic University of the Sacred Heart, L.go Gemelli 8, 00168 Rome, Italy; [2]Department of Human Pathology, Istituto Regina Elena, Rome, Italy; [3]Libero Istituto Universitario Campus Biomedico, Rome, Italy

**Summary** The aim of this study was to assess the association of p53 status with primary cytoreduction, response to chemotherapy and outcome in stage III–IV primary ovarian cancer patients. Immunohistochemical analysis of p53 was performed on formalin-fixed, paraffin-embedded specimens from 168 primary ovarian carcinomas by using the DO-7 monoclonal antibody. p53 nuclear positivity was found in 84 out of 162 (52%) malignant tumours. A higher percentage of p53 nuclear positivity was observed in patients with advanced stage of disease than in stage I–II (57% vs 23% respectively; $P = 0.0022$) and in poorly differentiated versus well/moderately differentiated tumours (59% vs 32% respectively; $P = 0.0038$). The multivariate analysis aimed to investigate the association of FIGO stage, grade and p53 status with primary cytoreduction in 136 stage III–IV patients showed that stage IV disease may influence the possibility to perform primary cytoreduction in ovarian cancer patients. p53-positivity also maintained a trend to be associated with poor chance of cytoreduction. In patients who underwent pathologic assessment of response, cases who did not respond to chemotherapy were much more frequently p53-positive than p53-negative (86% vs 14% respectively; $P = 0.012$). Moreover, patients with stage III disease and < 2-cm residual tumour were more likely to respond to treatment. In multivariate analysis, FIGO stage and p53 expression were independently correlated with pathologic response to chemotherapy. Time to progression and survival rates were shown not to be different in p53-positive versus p53-negative patients. © 1999 Cancer Research Campaign

**Keywords:** p53; ovarian cancer; cytoreduction; response to chemotherapy

Even though the bulk of experimental evidence has reported that aberrations of the tumour suppressor gene p53 play a critical role in ovarian carcinogenesis and progression (Marks et al, 1991; Kohler et al, 1993, Kupryjanczyk et al, 1993, 1994), we are still far from defining the potential usefulness of p53 assessment in the clinical management of ovarian cancer patients, especially in terms of prediction of response to chemotherapy and identification of patients at high versus low risk of progression, recurrence and death by disease (Marks et al, 1991; Hartmann et al, 1994; Henriksen et al, 1994; Klemi et al, 1995; Levesque et al, 1995; van der Zee et al, 1995; Diebold et al, 1996; Herod et al, 1996; Reles et al, 1996; Righetti et al, 1996; Buttitta et al, 1997; Dong et al, 1997; Geisler et al, 1997; Rohlke et al, 1997; Marx et al, 1998).

The difficulties, not exclusively limited to p53, in translating the information of biological factors from ‘in vitro’ models to the clinical setting in ovarian carcinoma, might come from the heterogeneous population of ovarian cancer patients examined in different studies; in particular, (a) the achievement of optimal tumour resection is differently defined, (b) there is no uniformity in chemotherapeutic regimens, and (c) response to chemotherapy, which is sometimes difficult to define given the high frequency of unmeasurable lesions, has been heterogeneously considered and analysed. The aim of this study was to investigate the association of p53 status with primary cytoreduction, response to chemotherapy and outcome in primary ovarian cancer patients. The study was conducted by analysing distinct homogeneous groups of patients, defined according to the type of primary surgery, extent of cytoreduction, criteria of response to chemotherapy and follow-up procedures, in order to minimize possible bias represented by inaccurate or unclearly defined selection of patients. Moreover, all surgical procedures, chemotherapeutic treatments and follow-up studies were performed by the same gynaecological oncology institution. For this study, we decided to utilize the immunohistochemical approach, since immunohistochemical results have been reported to be highly correlated with mutational analysis data (Marks et al, 1991; Bodner et al, 1992; Eccles et al, 1992; Kupryjanczyk et al, 1993) and immunohistochemistry is more suitable for routine utilization if a practical application of p53 assessment is to be achieved. The monoclonal antibody DO-7, which recognizes both the wild-type and the mutated form of p53 and has been shown to be the most suitable antibody for p53 assessment (Righetti et al, 1996) was used.

*Received 9 February 1999*
*Revised 19 April 1999*
*Accepted 22 April 1999*

*Correspondence to:* G Scambia

### PATIENTS AND METHODS

This study was conducted on 168 primary untreated ovarian cancer patients admitted to the Department of Gynecological

**Table 1** Clinico-pathological characteristics of ovarian cancer patients and distribution of p53-positivity

| Characteristics | No. (%) of patients | p53-positive cases No. (%) of patients | *P*-value[a] |
|---|---|---|---|
| All cases | 168 | 84 (50) | |
| LMP tumours | 6 (3.5) | 0 | |
| Carcinomas | 162 (96.5) | 84 (52) | |
| Age (years) | | | |
| ≤ 60 | 110 (68) | 55 (50) | 0.50 |
| > 60 | 52 (32) | 29 (56) | |
| Histotype | | | |
| Serous | 108 (66) | 56 (52) | |
| Mucinous | 6 (4) | 1 (17) | 0.26 |
| Endometrioid | 30 (19) | 17 (57) | |
| Undifferentiated | 14 (8.5) | 9 (64) | |
| Other | 4 (2.5) | 1 (25) | |
| FIGO stage | | | |
| I | 21 (13) | 5 (24) | |
| II | 5 (3) | 1 (20) | 0.0022[b] |
| III | 107 (66) | 60 (56) | |
| IV | 29 (18) | 18 (62) | |
| Grade of differentiation | | | |
| G1 | 16 (10) | 4 (25) | 0.0038[c] |
| G2 | 28 (17) | 10 (36) | |
| G3 | 104 (64) | 61 (59) | |
| NA | 14 (9) | – | |
| Ascites | | | |
| No | 71 (44) | 32 (45) | 0.15 |
| Yes | 91 (56) | 51 (57) | |

[a]Calculated by Fisher's exact test for proportion; [b]stage III–IV versus stage I–II; [c]grade 3 versus grade 1–2.

Oncology of the Catholic University of Rome, from 1988 to 1997. Clinico-pathologic characteristics of our patient population are summarized in Table 1. The flow-chart of our patient population subsets is shown in Figure 1. Since the study was aimed to assess the possible clinical role of p53 status in terms of association with ovarian tumour cytoreduction, response to chemotherapy and survival, most of the analyses were performed in the group of stage III–IV patients.

One hundred and thirty-six stage III–IV ovarian cancer patients underwent explorative laparotomy. Even though several cut-off values of residual tumour volume have been proposed, it has been reported that gradual gradations of residual disease can affect ovarian cancer patient prognosis (Potter et al 1991; Bristow et al, 1996; Heintz, 1996). Our patient population was divided into three subgroups according to the extent of residual disease: group A included 89 patients (65.5%) who were optimally (absent/microscopic) or sub-optimally (less than 2 cm residual disease) cytoreduced; group B included 15 (11%) patients with 2–5 cm residual disease; and Group C was constituted of 32 (23.5%) cases considered unresectable at the time of laparotomy and submitted only to multiple biopsies.

The 104 patients in which a measurable residual disease volume was left (group A and B) were considered for chemotherapy response assessment. Two to 3 weeks after surgery, four to six cycles of cisplatin-based chemotherapy (total cisplatin dose at least 400 mg $m^{-2}$) were administered to 102 (98%) patients (two patients were lost to follow-up). In only ten out of 102 patients (9.9%) the chemotherapy regimen included Taxol, making the separate statistical analysis of this subgroup unreliable. Gynaecological examination, abdominopelvic ultrasonography, CA-125 assay and radiological investigations, if necessary, were

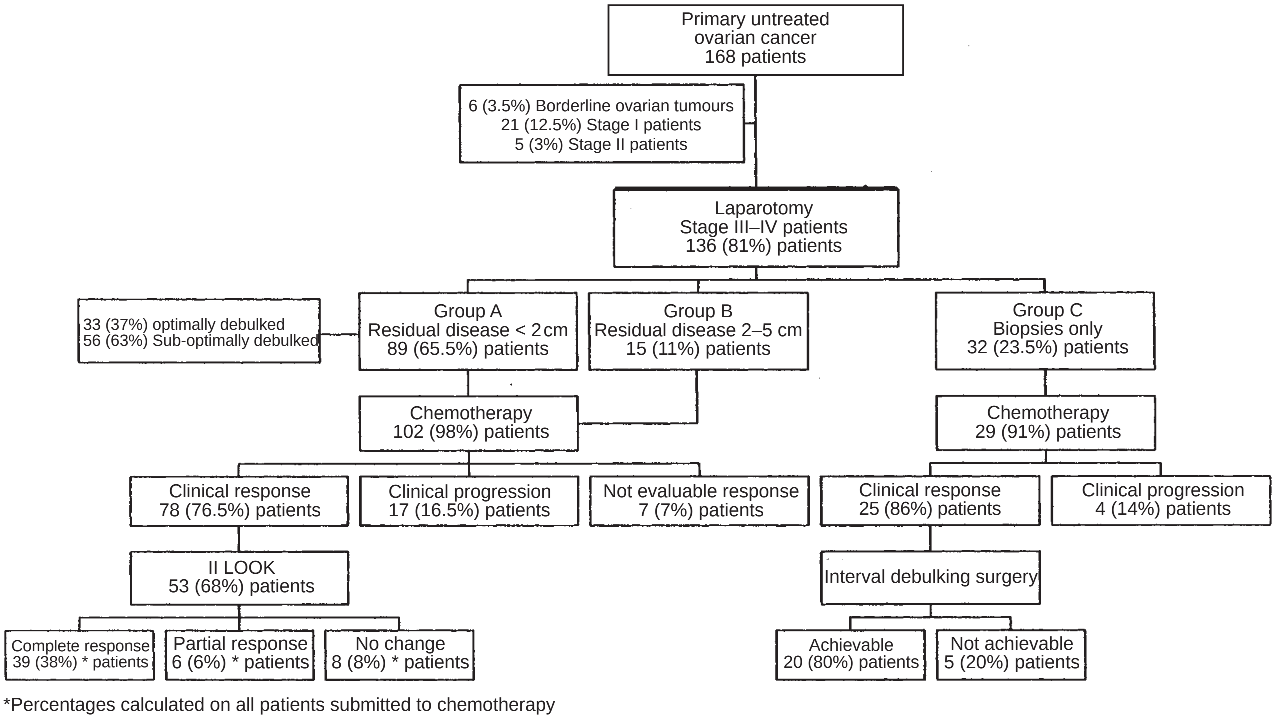

**Figure 1** Flow chart events of our patient population study

 *© 1999 Cancer Research Campaign*

performed monthly for the clinical assessment of response, which was recorded according to World Health Organization criteria (WHO, 1979).

Seven patients were not assessed for response to chemotherapy since they were lost to follow-up. Approximately 28 days after the last course (range 3–6 weeks), 53 patients out of 78 clinical responders underwent second-look laparoscopy or, in laparoscopy-negative cases, second-look laparotomy for the assessment of pathologic response. Second-look procedures were not performed in 25 patients because of patient refusal ($n$ = 17) and severe drug toxicity ($n$ = 8).

Peritoneal washings, careful inspection of the abdominal cavity and biopsy of all suspicious lesions were performed; in the case of no evidence of disease, at least 20 random biopsies were taken. Pathologic complete responders ($n$ = 39) received either no further therapy or alternative therapies and entered follow-up procedures. Pathological partial responders ($n$ = 6) and patients who showed pathologic stabilization/progression of disease ($n$ = 8) or clinically unresponsive to first-line treatment ($n$ = 17) were treated according to second-line chemotherapy regimens.

Unresectable patients (Group C) were submitted to two or three cycles of cisplatin-based chemotherapy before attempting a second cytoreductive effort which was limited to patients who showed clinical response to primary treatment ($n$ = 25).

### Immunohistochemical analysis

Immunohistochemical analysis of the p53 protein was performed on 168 ovarian carcinomas, using the avidin–biotin–peroxidase complex method (ABC) (Dako, Carpinteria, CA, USA) and the specific mouse monoclonal antibody for p53 (DO-7, Dako, Carpinteria, CA, USA) which recognizes both the wild-type and the mutant form of p53 protein.

Five-micrometer sections were dewaxed in xylene, rehydrated in descending concentration of alcohol down to 80%, washed in tap and distilled water, and then treated with 0.3% hydrogen peroxide in methanol for 25 min to remove endogenous peroxidase activity. Sections were washed in Tris-buffered saline solution (TBS) (pH 7.6) and then microwave-treated (two cycles of 5 min) in 10 mM trisodium citrate buffer solution (pH 6.0). After three washes in TBS, sections were incubated with normal serum as blocking reagent to minimize non-specific binding.

A 1:100 dilution of the specific mouse monoclonal antibody to p53 protein (DO-7) was applied for 60 min at room temperature. The sections were then incubated with the biotinylated goat anti-mouse IgG and with avidin–biotin–peroxidase complex for 10 min at room temperature. Finally, the sections were washed in TBS, stained by incubation with 3-amino-9-ethylcarbazole (AEC) (Dako, Carpinteria, CA, USA) for 10–20 min and then counter-stained with haematoxylin. Fetal calf serum (Dako, Carpinteria, CA, USA) was used as a negative control.

The sections were examined independently by two observers (MM and AF) by means of light-microscopic examination and scored according to the intensity of staining and proportion of cells stained. In case of disagreement, the sections were reviewed on a two-headed microscope (Laborlux-S, Leitz), in order to reach a consensus. In detail, the cases were scored as negative when no staining was observed. When the positivity was observed in more than 1% of tumour cells (which corresponds to the median value), the cases were scored as positive.

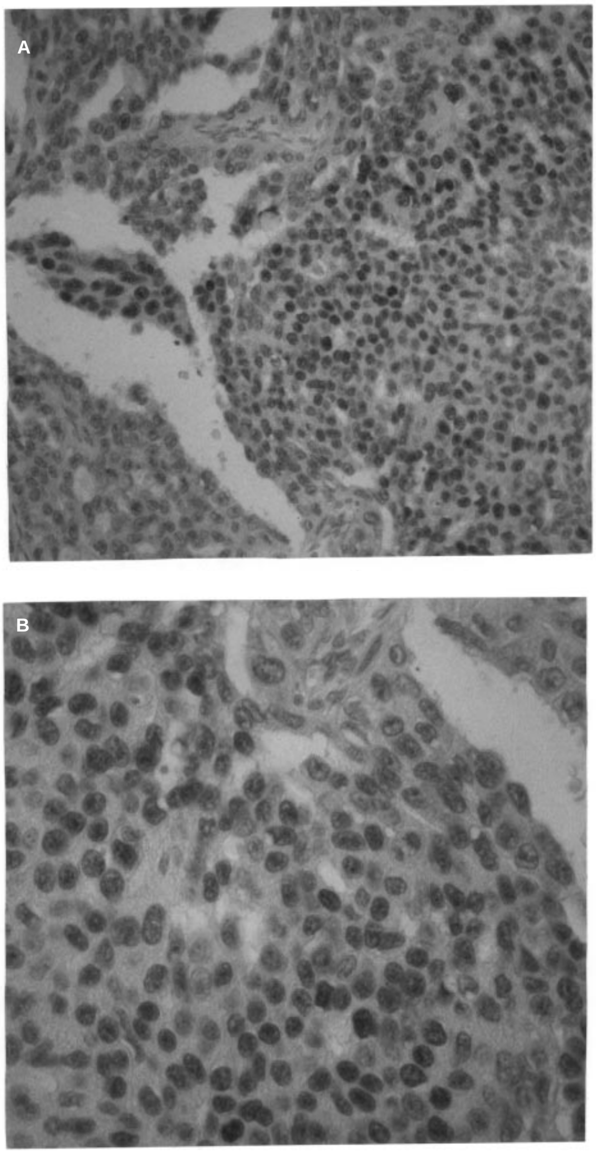

**Figure 2** p53 nuclear immunostaining in ovarian cancer tissue. (**A**) Original magnification 40×. (**B**) Original magnification 100×

The scoring of the immunohistochemical data was determined without any knowledge of the clinico-pathological parameters or of the follow-up data.

### Statistical analysis

Fisher's exact test for proportion was used to analyse the distribution of p53-positivity according to clinico-pathological characteristics.

The association of clinico-pathological parameters and p53-status with response to chemotherapy and cytoreduction was assessed in a multivariate model by the stepwise logistic regression (Cox, 1970). This model enabled us to predict the probability of a particular outcome to take place, after combining the informations deriving from several parameters. In this model, the β-estimate corresponds to the 'regression coefficient' for each variable, and the $\chi^2$ value indicates the strength of the evidence against the null hypothesis of no association between a specific

**Table 2** Distribution of clinico-pathological parameters and p53 status according to residual tumour volume in stage III–IV ovarian cancer patients

| Characteristics | Group A No. (%) of patients | Group B+C No. (%) of patients | *P*-value[a] |
|---|---|---|---|
| All cases | 89 | 47 | |
| Age (years) | | | |
| ≤60 | 61 (68.5) | 30 (64) | 0.70 |
| > 60 | 28 (31.5) | 17 (36) | |
| Histotype | | | |
| Serous | 61 (68.5) | 31 (66) | |
| Mucinous | 2 (2) | 1 (2) | |
| Endometrioid | 20 (22) | 6 (13) | 0.20[b] |
| Undifferentiated | 5 (5.5) | 8 (17) | |
| Other | 1 (1) | 1 (2) | |
| FIGO stage | | | |
| III | 79 (89) | 28 (59.5) | 0.0002 |
| IV | 10 (11) | 19 (40.5) | |
| Grade of differentiation | | | |
| G1-G2 | 24 (28.5) | 5 (12.5) | 0.068 |
| G3 | 60 (71.5) | 35 (87.5) | |
| Ascites | | | |
| No | 35 (40) | 13 (28) | 0.22 |
| Yes | 54 (60) | 34 (72) | |
| p53 status | | | |
| Negative | 43 (48) | 15 (32) | 0.07 |
| Positive | 46 (52) | 32 (68) | |

[a]Calculated by Fisher's exact test for proportion; [b]calculated by $\chi^2$ test (not accurate).

**Table 3** Distribution of clinico-pathological parameters and p53 status according to pathological response to chemotherapy in stage III–IV ovarian cancer patients

| Characteristics | Pathological complete response No. (%) of patients | Pathological partial/no change response No. (%) of patients | *P*-value[a] |
|---|---|---|---|
| All cases | 39 | 14 | |
| Age (years) | | | |
| ≤ 60 | 36 (92) | 11 (78.5) | 0.32 |
| > 60 | 3 (8) | 3 (21.5) | |
| Histotype | | | |
| Serous | 28 (72) | 9 (64) | 0.68 |
| Endometrioid | 6 (15) | 4 (29) | |
| Undifferentiated | 4 (10) | 1 (7) | |
| Other | 1 (3) | 0 | |
| FIGO stage | | | |
| III | 36 (92) | 8 (57) | 0.006 |
| IV | 3 (8) | 6 (43) | |
| Grade of differentiation | | | |
| G1–G2 | 9 (25) | 6 (46) | 0.17 |
| G3 | 27 (75) | 7 (54) | |
| Ascites | | | |
| No | 15 (38) | 6 (43) | 1.0 |
| Yes | 23 (62) | 8 (57) | |
| Residual tumour | | | |
| Group A | 36 (92) | 9 (64) | 0.023 |
| Group B | 3 (8) | 5 (36) | |
| p53 status | | | |
| Negative | 21 (54) | 2 (14) | 0.012 |
| Positive | 18 (46) | 12 (86) | |

[a]Calculated by Fisher's exact test for proportion.

variable and the outcome. The probability of obtaining a $\chi^2$ value greater than above is known as *P*-value.

All medians and life-tables were computed using the product-limit estimate and the curves were examined by means of the log-rank test (Mantel 1966). Time to progression and overall survival were calculated from the day of the first surgery to the date of clinical or pathological progression or death. All survival analyses were performed using SOLO Statistical Software (BMDP Statistical Software Inc., Los Angeles, CA, USA).

### RESULTS

Figure 2 shows a representative example of p53 immunohistochemical analysis in ovarian cancer tissue. Nuclear immunostaining was evident in many cancer cells, whereas the stromal component was unreactive.

Table 1 shows the distribution of p53-positivity in six low malignant potential (LMP) tumours and 162 primary ovarian carcinomas. All LMP tumours were p53-negative, while the percentage of p53 nuclear positivity was 52% in the group of malignant tumours.

In Table 1 the distribution of p53-positivity according to clinico-pathological characteristics is shown. p53-positivity was not distributed differently according to patient age and histotype. On the other hand, a higher percentage of p53 nuclear positivity was observed in patients with advanced stage of disease than in stage I–II (57% vs 23% respectively; $P = 0.0022$). Similarly, poorly differentiated tumours were more frequently p53-positive than well/moderately differentiated ones (59% vs 32% respetively; $P = 0.0038$). No association between p53 status and absence/presence of ascites was found.

The association of clinico-pathological features and p53 status with primary cytoreduction, response to chemotherapy and survival was focused on the subgroup of stage III–IV patients ($n = 136$), as pointed out in Materials and Methods.

In order to investigate the possible independent role of clinico-pathological features and p53 status as parameters associated with primary cytoreduction in the multivariate model, the dichotomization of patients according to the extent of residual tumour volume had to be performed. Analyses were conducted by comparing group A+B versus C, or alternatively group A versus group B+C. Since similar results were obtained in both comparisons, we decided to show the analysis comparing subgroup of patients with tumour volume < 2 cm (group A) versus ≥ 2 cm (group B+C) (Table 2), due to the reason that the value of 2-cm residual disease is the most frequently used cut-off for primary debulking surgery likely to have a relevant impact on patients' survival.

A higher percentage of stage III with respect to stage IV cases was observed in the group A (89% vs 11% respectively; $P = 0.0002$). Moreover, a trend towards a higher percentage of poorly differentiated compared to well/moderately differentiated tumours in group B+C was observed ($P = 0.068$). Table 2 also shows that a higher percentage of p53-positive than p53-negative cases was found in the group B+C (68% vs 32% respectively; $P = 0.07$). Moreover, within group A, no differences in p53-positivity according to the amount of residual tumour was found: p53-positivity was observed in 14 out of 33 patients (42%) with absent or microscopic residual tumour and in 32 out of 56 patients (57%) with < 2-cm residual tumour (data not shown).

© 1999 Cancer Research Campaign

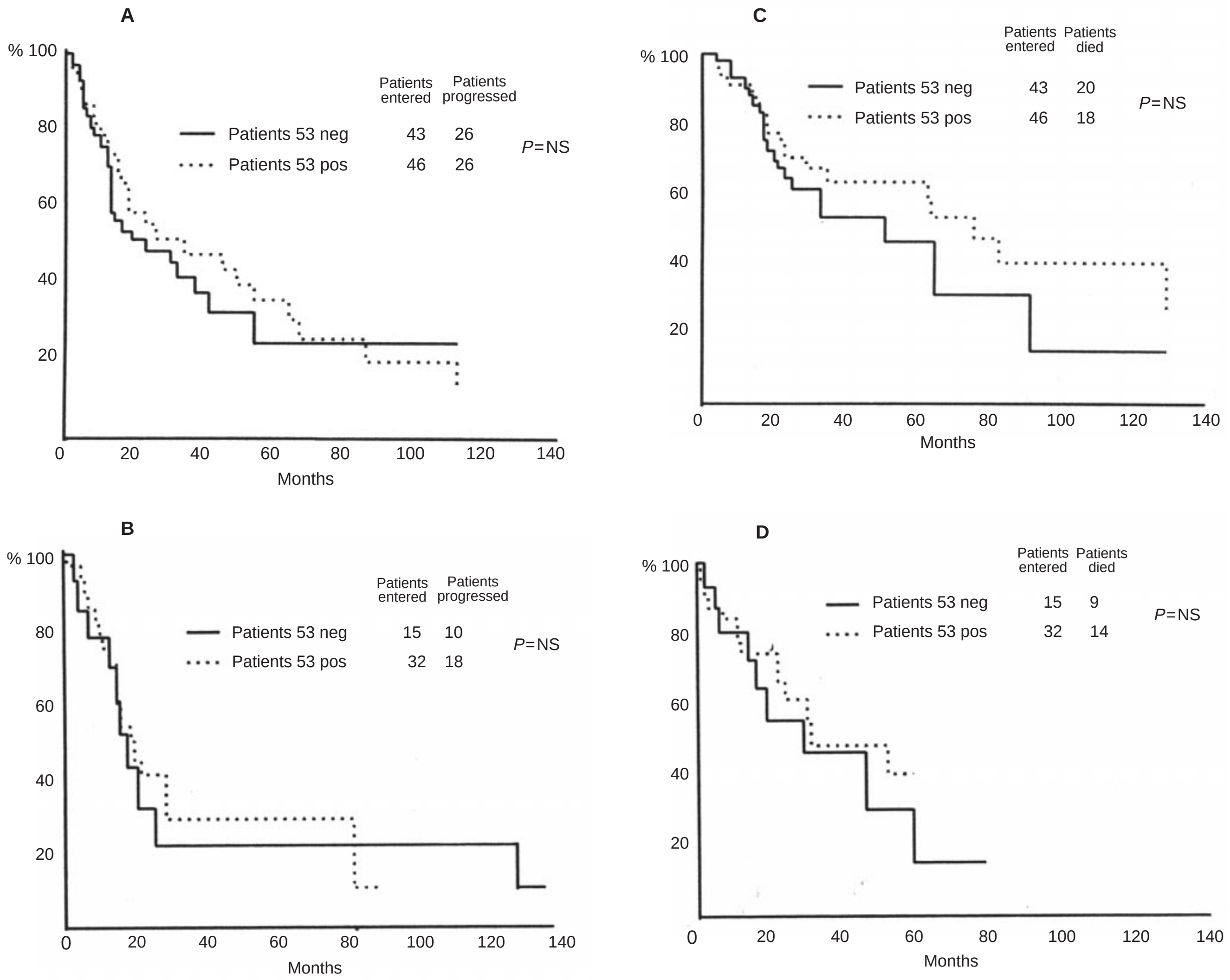

**Figure 3** Survival analysis according to p53 status in advanced ovarian cancer patients. (**A**) Time to progression in group A; (**B**) time to progression in group B+C; (**C**) overall survival in group A; (**D**) overall survival in group B+C

The multivariate analysis used to investigate the association of FIGO stage, grade and p53 status with primary cytoreduction was carried out. Stage IV disease was demonstrated to be strongly associated with poor chance of primary cytoreduction (β-estimate: 1.79; $\chi^2$: 13.66; *P*-value: 0.0002) and p53-positivity also maintained the trend to be associated with low possibility of cytoreduction (β-estimate: 0.80; $\chi^2$: 3.38; *P*-value: 0.065). The $\chi^2$ of the model was 19.12 (2 degrees of freedom) with a *P*-value of 0.0001.

The possible correlation of clinico-pathological characteristics as well as p53 status with response to chemotherapy was analysed in group A and B patients, after dichotomizing type of response into pathological complete response ($n$ = 39) versus pathological partial or no response to treatment ($n$ = 14) plus clinical progression ($n$ = 17). No association between p53 status and response to chemotherapy was observed (data not shown). However, when the analysis was performed only in patients who underwent pathological assessment of response ($n$ = 53) (Table 3), patients who did not respond to chemotherapy were more frequently p53-positive than p53-negative (86% vs 14% respectively; $P$ = 0.012). Moreover, stage III patients were more likely to respond to treatment than stage IV cases (92% vs 8% respectively; $P$ = 0.006). Finally, group A patients were found to have a higher chance of response than group B patients (92% vs 8% respectively; $P$ = 0.023). Age, histotype, grade of differentiation and presence/absence of ascites did not show any association with response to chemotherapy.

The multivariate analysis, including the variables significantly associated with response to chemotherapy in univariate analysis, showed that FIGO stage (β-estimate: 2.41; $\chi^2$: 6.58; *P*-value: 0.01) and p53 expression (β-estimate: 2.13; $\chi^2$: 5.22; *P*-value: 0.022) were independently correlated with pathological response to chemotherapy. The $\chi^2$ of the model was 15.04 (2 degrees of freedom) with a *P*-value of 0.0005.

In the group of unresectable patients (group C), assessment of response to 2–3 cycles of chemotherapy was evaluated on 29/32 cases (three patients died before starting chemotherapy). Nine patients who did not show any clinical benefit from treatment or proved not to be susceptible to interval debulking surgery (IDS) were compared to patients in which IDS was achievable ($n$ = 20). Neither the clinico-pathological parameters nor the p53 status were shown to be differently distributed in these two groups (data not shown).

© 1999 Cancer Research Campaign

**Table 4** Studies analysing the association between p53 status, response to chemotherapy and prognosis in ovarian cancer patients

| Author (year) | Antibody (sections) | No. of patients | No. of stage I–II | No. of optimally debulked | Response to chemotherapy | No. of pathological complete response | Association with poor response | Multivariate analysis for response | Association with poor survival (population analysed) | Multivariate analysis for survival |
|---|---|---|---|---|---|---|---|---|---|---|
| Marks et al (1991) | PAb 1801 (frozen) | 107[a] | 15 | 15[b] | – | – | – | – | NS | – |
| Hartmann et al (1994) | PAb 1801 (paraffin) | 284 | 56 | – | 238 | 78 | NS | – | Direct (P = 0.04) all stages | NS |
| Henriksen et al (1994) | PAb 1801 (frozen) | 55 | 26 | 31[c] | – | – | – | – | Direct all stages | – |
| Klemi et al (1995) | n.d. (paraffin) | 136 | 47 | 68[d] | – | – | – | – | Direct (P = 0.002) all stages | Direct |
| Levesque et al (1995) | Pab 240 (ELISA) | 90 | 27 | 34[e] | – | – | – | – | Direct (P = 0.06) all stages | NS |
| van der Zee et al (1995) | CM1 (paraffin) | 89[f] | 20 | 50[d] | 70 | 21 | NS[g] | – | Direct (P < 0.001) stage III–IV | NS |
| Diebold et al (1996) | DO-1 (paraffin) | 148[h] | 71 | – | – | – | – | – | Direct all stages and LMP | Direct |
| Herod et al (1996) | PAb 1801 (paraffin) | 701 | 3 | 27[d] | 64[i] | – | NS | – | NS | Direct (border-line value) |
| Reles et al (1996) | BP53-12-1, DO-1 (paraffin) | 179 | 74 | 145[d] | – | – | – | – | NS | – |
| Righetti et al (1996) | DO-7 (paraffin) | 33 | – | 8[d] | 32 | 13[l] | Direct | – | – | – |
| Buttitta et al (1997) | PAb 1801 (frozen) | 53 | 14 | – | 33 | 7 | Direct | – | Direct[m] not clearly specified | – |
| Dong et al (1997) | DO-7 (paraffin) | 141[n] | 47 | – | 44 | – | Direct[o] | – | Direct (P = 0.003) all stages | NS |
| Geisler et al (1997) | PAb 1801 (frozen) | 83 | 16 | 56[b] | – | – | – | – | Direct[m] (P = 0.001) not specified | Direct[p] |
| Rohlke et al (1997) | Ab-6 (paraffin) | 104[q] | 28 | – | 50 | 35 | NS | – | Direct all stages, stage III–IV | Direct |
| Marx et al (1998) | DO-7 (paraffin) | 187 | 40 | 5[r] | – | – | – | – | Direct all stages | – |
| Current study | DO-7 (paraffin) | 162 | 26 | 89[d,s] | 95 | 39 | Direct[t] | Direct | NS stage III–IV | – |

[a]38 pretreated patients; [b]less than 1 cm; [c]less than 2 cm; [d]55 patients received surgery in the referring hospital; [e]number of pathologically complete responses not specified; [f]P = 0.014, when complete response vs other response types was compared; [g]residual tumour = absent; all stages included; [h]LMP tumours included (*n* = 30); [i]treatment and subgroups not clearly defined; [l]pathological response (*n* = 12); [m]Kaplan and Meier analysis not performed; [n]including 16 LMP; [o]response was clinically defined; poor response was associated with negative or high p53 expression; [p]logistic regression analysis was used; [q]89 out 136 stage III–IV patients; [r]in pathologically verified response.

### Survival analysis

Survival analysis was focused on the more homogeneous group of stage III–IV ovarian cancer patients. The median follow-up period was 22 months (range 1–141 months), during which we observed 80 progressions and 61 deaths of disease. The median time to progression was 15 months (range 1–126 months). Time to progression (Figure 3 A, B) and overall survival rates (Figure 3 C, D) were not different in p53-positive versus p53-negative patients.

Survival analysis gave superimposable results when performed on the entire population (data not shown).

### DISCUSSION

At present, few studies have investigated the association of p53 status with primary cytoreduction in ovarian carcinoma. In stage III–IV ovarian cancer patients, a higher percentage of p53-positivity was found in the group of patients with a larger extent of residual disease. The risk that the feasibility of tumour reduction, and the extent of cytoreduction might be influenced by the skills of different surgeons or involvement of non-gynaecological oncology surgeons is minimal, since all cases analysed were submitted to explorative laparotomies and cytoreductive efforts by the same surgical team. Moreover, the association of p53-positivity with advanced stage of disease or other clinical parameters of tumour aggressiveness appears not to be the entire explanation to our findings, since FIGO stage as well as p53 status were found to be independently associated with ovarian tumour cytoreduction as determined by multivariate analysis.

A common finding of gynaecological oncology surgeons is that, besides the simple bulky extension of tumour mass, the extent to which tumour permeates and invades crucial anatomic structures like mesenteries and vascular supplies could heavily influence the possibility of successful tumour debulking. In this context, the observation by Bouvet et al (1998) that the reintroduction of the wild-type p53 gene into human colon cancer cells is associated with the inhibition of neo-vascularization in vivo seems quite intriguing, suggesting that abnormal p53 expression could also be

*© 1999 Cancer Research Campaign*

involved in the deregulation of neo-angiogenesis and/or tumour angiotropism.

Even though the statistical significance of our finding is apparently not strong enough to support the clinical usefulness of p53 status assessment in predicting the feasibility of primary tumour cytoreduction, it is conceivable that tumour susceptibility to be cytoreduced may also be influenced by intrinsic biological characteristics of aggressiveness, thus emphasizing the importance of simultaneous investigation of multiple biological factors as possible predictors of successful tumour debulking.

We observed that a higher percentage of p53-positive cases was associated with a poor chance of response to treatment since patients who did not respond to chemotherapy were more frequently p53-positive. On the other hand, a certain number of cases who achieved pathological response to treatment were found to show p53 nuclear positivity. This findings, which confirms previous data by Righetti et al (1996), could be explained by the fact that wild-type p53 protein can accumulate without detectable mutations, and this may be due to altered regulation of p53 expression or presence of p53-interacting and -stabilizing proteins (Momand et al, 1992).

Several in vitro observations (Brown et al, 1993; Gjerset et al, 1993; Perego et al, 1996) have suggested an involvement of p53 protein in determining the response to cisplatin-based chemotherapy. Results of previous reports dealing with the association of p53 with response to chemotherapy in ovarian carcinoma patients are conflicting (Table 4). Our results, confirmed by multivariate analysis taking into account other relevant clinico-pathological parameters, are in agreement with the observations of previous studies (Righetti et al, 1996; Buttitta et al, 1997; Dong et al, 1997) and some recent findings (Katsaros et al, 1998). In the study by van der Zee et al (1995) p53-positive cases have been reported not to be distributed differently according to type of response. However, after dichotomizing complete response versus no response, a statistically significant higher percentage of p53-positivity was found to be associated with a poor chance of response to treatment. Therefore, five out of eight studies demonstrated that p53 expression is associated with response to cisplatin-based chemotherapy. Moreover, a significant dose–response effect has been recently reported in p53-negative versus p53-positive ovarian cancer patients (Marx et al, 1998).

Our finding that p53 status fail to be correlated with response to chemotherapy when patients experiencing clinical progression were included in the analysis, seemed quite surprising. A possible explanation of this observation may be that clinically unresponsive patients have tumours with clinico-pathological and biological characteristics of aggressiveness which could cause p53 status to become of minor importance. In particular, in our series, clinically progressive patients were shown to be frequently older in age and their tumours were more often poorly differentiated with respect to other patient subgroups. Moreover, the same subpopulation had a higher percentage of epidermal growth factor receptor positivity (unpublished observations), which has previously been demonstrated to negatively affect survival and response to chemotherapy in ovarian cancer patients (Scambia et al, 1995). In addition, alterations of critical molecules downstream of wild-type p53 protein could occur, which are responsible for deregulating p53-promoted apoptosis pathways and can influence response to treatment (Momand et al, 1992; Michieli et al 1994; Werness et al, 1997). We failed to find any association between p53 status and clinical outcome in terms of time to progression and overall survival, as reported by other authors (Marks et al 1991; Herod et al, 1996; Reles et al, 1996). However, as summarized in Table 4, results that were not always examined in multivariate setting are quite conflicting, and populations included were not superimposable. Moreover, it has to be considered that a high percentage of our stage III–IV ovarian cancer patients were submitted to second-line or very high-dose chemotherapy regimens which could affect prognosis independently of p53 status. These observations could be a reasonable explanation of the still growing controversy.

In conclusion, our study reported an interesting association between p53 status and primary ovarian cancer cytoreduction suggesting that the possible role of p53 in influencing successful tumour debulking deserves further attention. The association of p53 with response to cisplatin-based chemotherapy has also been confirmed in multivariate analysis. Finally the need to precisely define patient subpopulations from a clinical point of view in order to provide a meaningful evaluation of the clinical significance of biologic factors has been emphasized.

### ACKNOWLEDGEMENT

This work was partially supported by the Italian Association for Cancer Research (AIRC).

### REFERENCES

Bodner SM, Minna JD, Jensen SM, D'Amico D, Carbone D, Mitsudomi T, Fedorko J, Buchhagen DL, Nau MM and Gazdar AF (1992) Expression of mutant p53 proteins in lung cancer correlates with the loss of p53 gene mutation. *Oncogene* **7**: 743–749

Bouvet M, Ellis LM, Nishizaki M, Fijiwara T, Tiu W, Bucana CD, Fang B, Lee JJ and Roth J (1998) Adenovirus-mediated wild-type p53 gene transfer down regulates vascular endothelial growth factor expresion and inhibits angiogenesis in human colon cancer. *Cancer Res* **58**: 2288–2292

Bristow RE, Lagasse LD, Karlan BY (1996) Secondary surgical cytoreduction for advanced epithelial ovarian cancer. *Cancer* **78**: 2049–2062

Brown R, Clugston C, Burns P, Edlin A, Vasey P, Kaye SB and Vojtesek B (1993) Increased accumulation of p53 protein in cisplatin-resistant ovarian cell lines. *Int J Cancer* **55**: 678–684

Buttitta F, Marchetti A, Gadducci A, Pellegrini S, Morganti M, Carnicelli V, Cosio S, Gaggetti O, Genazzani AR and Bevilacqua G (1997) p53 alterations are predictive of chemoresistance and aggressiveness in ovarian carcinomas: a molecular and immunohistochemical study. *Br J Cancer* **75**: 230–235

Cox DR (1970) *Analysis of Binary Data*. Methuen: London

Diebold J, Baretton G, Felchner M, Meier W, Dopfer K, Schmidt M and Lohrs U (1996) Bcl-2 expression, p53 accumulation and apoptosis in ovarian carcinomas. *Am J Clin Pathol* **105**: 341–349

Dong Y, Walsh MD, McGuckin MA, Cummings MC, Gabrielli BG, Wright GR, Hurst T, Khoo SK and Parsons PG (1997) Reduced expression of retinoblastoma gene product (pRB) and high expression of p53 are associated with poor prognosis in ovarian cancer. *Int J Cancer* **74**: 407–414

Eccles DM, Brett L, Lessels A, Gruber L, Lane D, Steel CM and Leonard RC (1992) Overexpression of the p53 protein and allele loss at 17p13 in ovarian carcinoma. *Br J Cancer* **65**: 40–44

Geisler JP, Geisler HE, Wiemann MC, Givens SS, Zhou Z and Miller GS (1997) Quantification of p53 in epithelial ovarian cancer. *Gynecol Oncol* **66**: 435–438

Gjerset RA, Turla ST, Sobol RE, Scalise JT, Mercola D, Collins M and Hopkins PJ (1993) Use of wild type p53 to achieve complete treatment sensitization of tumor cells expressing endogenous mutant p53. *Mol Carcinog* **14**: 275–285

Hartmann LC, Podratz KC, Keeney GL, Kamel NA, Edmonson JH, Grill JP, Su JQ, Katzmann J and Roche PC (1994) Prognostic significance of p53 immunostaining in epithelial ovarian cancer. *J Clin Oncol* **12**: 64–69

Heintz APM (1996) Surgery in ovarian cancer: the concept of cytoreductive surgery. *Curr Opin Obst Gynecol* **8**: 8–11

Henriksen R, Strang P, Wilander E, Backstrom T, Tribukait B and Oberg K (1994) p53 expression in epithelial ovarian neoplasms: relationship to clinical and pathological parameters, Ki-67 expression and flow-cytometry. *Gynecol Oncol* **53**: 301–306

© 1999 Cancer Research Campaign 

Herod JJO, Eliopoulos AG, Warwick J, Niedobitek G, Kern DJ and Young LS (1996) The prognostic significance of Bcl-2 and p53 expression in ovarian carcinoma. *Cancer Res* **56**: 2178–2184

Katsaros D, Levesque M, Genta F, Durando A, Arisio R, Yu H, Diamandis EP and Massobrio M (1998) Prognostic and predictive value of p53 and waf-1 expression in epithelial ovarian cancer. *Proc Am Ass Cancer Res* **39** (abstract 1355)

Klemi PJ, Pylkkanen L, Kiilholma P, Kurvinen K and Joensuu H (1995) p53 protein detected by immunohistochemistry as a prognostic factor in patients with epithelial ovarian carcinoma. *Cancer* **76**: 1201–1208

Kohler MF, Kerns BM, Humphrey PA, Marks JR, Bast RC and Berchuck A (1993) Mutation and overexpression of p53 in early stage epithelial ovarian cancer. *Obstet Gynecol* **81**: 643–650

Kupryjanczyk J, Bell DA, Yandell DW, Scully RE, Thor AD (1994) p53 expression in ovarian borderline tumors and stage I carcinomas. *Am J Clin Pathol* **102**: 671–676

Kupryjanczyk J, Thor A, Beauchamp R, Merritt V, Edgerton SM, Bell DA and Yandell DW (1993) p53 gene mutations and protein accumulation in human ovarian cancer. *Proc Natl Acad Sci USA* **90**: 4961–4965

Levesque MA, Katsarods D, Yu H, Zola P, Sismondi P, Giardina G and Diamandis EP (1995) Mutant p53 protein overexpression is associated with poor outcome in patients with well or moderately differentiated ovarian carcinoma. *Cancer* **75**: 1327–1338

Mantel N (1966) Evaluation of survival data and two new rank order statistics arising its consideration. *Cancer Chemother Rep* **50**: 163–170

Marks JR, Davidoff AM, Kerns BJ, Humphrey PA, Pence JC, Dodge RK, Clarke-Pearson DL, Iglehart JD, Bast RC and Berchuck A (1991) Overexpression and mutation of p53 in epithelial ovarian cancer. *Cancer Res* **51**: 2979–2984

Marx D, Meden H, Ziemek T, Lenthe T, Kuhn W and Schauer A (1998) Expression of the p53 tumour suppressor gene as prognostic marker in platinum treated patients with ovarian cancer. *Eur J Cancer* **34**: 845–850

Michieli P, Chedid M, Lin D, Pierce JM, Mercer WE and Givol D (1994) Induction of waf1/cip1 by a p53-independent pathway. *Cancer Res* **54**: 3391–3395

Momand J, Zambetti GP, Olson DC, Gorge D and Levine HJ (1992) The mdm-2 oncogene product forms a complex with the p53 protein and inhibits p53-mediated transactivation. *Cell* **69**: 1237–1245

Perego P, Giarola M, Righetti SC, Supino R, Cesarini C, Delia D, Pierotti MA, Miyashita T, Reed JC and Zunino F (1996) Association between cisplatin resistance and mutation of p53 gene and reduced bax expression in ovarian carcinoma cell systems. *Cancer Res* **56**: 556–562

Potter ME, Partridge EE, Hatch KD, Soong SJ, Austin JM and Shingleton HM (1991) Primary surgical therapy of ovarian cancer: how much and when. *Gynecol Oncol* **40**: 195–200

Reles A, Schmider A, Press MF, Schonborn I, Friedmann W, Huber-Schumacher S, Stronmeyer T and Lintenegger W (1996) Immunostaining of p53 protein in ovarian carcinoma: correlation with histopathological data and clinical outcome. *J Cancer Res Clin Oncol* **122**: 489–494

Righetti, Della Torre G, Pilotti S, Menard S, Ottone F, Colnaghi MI, Pierotti MA, Lavarino C, Oriana S, Cornarotti M, Bohm S, Bresciani GC, Spatti G and Zunino F (1996) Comparative study of p53 gene mutations, protein accumulation, and response cisplatin-based chemotherapy in advanced ovarian carcinoma. *Cancer Res* **56**: 689–693

Rohlke P, Milde-Langosch K, Weyland C, Pichlmeier U, Janet W and Loning T (1997) p53 is a persistent and predictive marker in advanced ovarian carcinomas: multivariate analysis including comparison with Ki67 immunoreactivity. *J Cancer Res Clin Oncol* **123**: 496–501

Scambia G, Benedetti-Panici P, Ferrandina G, Distefano M, Salerno G, Romanini ME, Fagotti A and Mancuso S (1995) Epidermal growth factor, oestrogen and progesterone receptor expression in primary ovarian cancer: correlation with clinical outcome and response to chemotherapy. *Br J Cancer* **72**: 361–366

van der Zee AGJ, Hollema H, Suurmeijer AJH, Krans M, Sluiter WJ, Willanse PH, Alders JG and de-Vries EF (1995) Value of p-glycoprotein, glutathione S-transferase pi, c-erbB-2, and p53 as prognostic factors in ovarian carcinomas. *J Clin Oncol* **13**: 70–78

Werness BA, Jobe JS, DiCioccio A and Pver S (1997) Expression of the p53 induced tumor suppressor $p21^{waf1/cip1}$ in ovarian carcinomas: correlation with p53 and Ki-67 immunohistochemistry. *Int J Gynecol Pathol* **16**: 149–155

World Health Organization (1979) *WHO Handbook for Reporting Results of Cancer Treatment*. WHO: Geneva, pp. 16–21

 *© 1999 Cancer Research Campaign*
